# Functional Validation of Candidate Genes Detected by Genomic Feature Models

**DOI:** 10.1534/g3.118.200082

**Published:** 2018-03-08

**Authors:** Palle Duun Rohde, Solveig Østergaard, Torsten Nygaard Kristensen, Peter Sørensen, Volker Loeschcke, Trudy F. C. Mackay, Pernille Sarup

**Affiliations:** * Center for Quantitative Genetics and Genomics, Department of Molecular Biology and Genetics; ‡ Center for Integrative Sequencing; § Section for Genetics, Ecology and Evolution, Department of Bioscience, Aarhus University, 8830 Tjele, Denmark; † The Lundbeck Foundation Initiative for Integrative Psychiatric Research, *i*PSYCH, 8000 Aarhus, Denmark; ** Section for Biology and Environmental Science, Department of Chemistry and Bioscience, Aalborg University, 9220 Aalborg, Denmark; †† Department of Biological Sciences; ‡‡ Program in Genetics; §§ W. M. Keck Center for Behavioral Biology, North Carolina State University, Raleigh, North Carolina 27695

**Keywords:** *Drosophila melanogaster*, DGRP, genomic prediction, set test, locomotor activity

## Abstract

Understanding the genetic underpinnings of complex traits requires knowledge of the genetic variants that contribute to phenotypic variability. Reliable statistical approaches are needed to obtain such knowledge. In genome-wide association studies, variants are tested for association with trait variability to pinpoint loci that contribute to the quantitative trait. Because stringent genome-wide significance thresholds are applied to control the false positive rate, many true causal variants can remain undetected. To ameliorate this problem, many alternative approaches have been developed, such as genomic feature models (GFM). The GFM approach tests for association of set of genomic markers, and predicts genomic values from genomic data utilizing prior biological knowledge. We investigated to what degree the findings from GFM have biological relevance. We used the *Drosophila* Genetic Reference Panel to investigate locomotor activity, and applied genomic feature prediction models to identify gene ontology (GO) categories predictive of this phenotype. Next, we applied the covariance association test to partition the genomic variance of the predictive GO terms to the genes within these terms. We then functionally assessed whether the identified candidate genes affected locomotor activity by reducing gene expression using RNA interference. In five of the seven candidate genes tested, reduced gene expression altered the phenotype. The ranking of genes within the predictive GO term was highly correlated with the magnitude of the phenotypic consequence of gene knockdown. This study provides evidence for five new candidate genes for locomotor activity, and provides support for the reliability of the GFM approach.

One of the major challenges in modern biology is to understand the link between molecular genetic variation and quantitative trait variation. For the vast majority of quantitative traits and diseases, phenotypic variation is caused by the joint effects of multiple segregating genetic variants, their interactions, environmental effects, and genotype-environment interactions and correlations (Falconer and Mackay 1996; Lynch and Walsh 1998). Knowledge of the genetic architecture of complex traits including species-specific causal genetic variants, and the distribution of their effect sizes and frequencies is important in multiple disciplines, such as animal and plant breeding, adaptive evolution, and in the study of complex human diseases and disorders.

Technological advancements in molecular biology, in particular the development of array-based and high-throughput sequencing platforms, have enabled large scale genome-wide scans for statistical associations between single nucleotide polymorphisms (SNPs) and quantitative traits and diseases (Balding 2006; Hardy and Singleton 2009). These genome-wide association studies (GWAS) have now been conducted on a large range of human diseases and traits, livestock and plant production traits (Dekkers 2012; Xiao *et al.* 2017), model organisms (Atwell *et al.* 2010; Mackay *et al.* 2012), as well as non-model species (Husby *et al.* 2015).

One of the major challenges with GWAS is the inability to detect all causal SNPs, or all SNPs correlated with the causal variants. Stringent genome-wide significance thresholds are needed in order to efficiently control the false positive rate. Because most SNP effect sizes are small to moderate, the majority of the causal variants will remain undetected (Yang *et al.* 2010, 2011; Visscher *et al.* 2012). Therefore, in recent years, methods have been developed for assessing the joint effect of multiple SNPs on trait variability. Such methods include set test approaches (Mooney *et al.* 2014; de Leeuw *et al.* 2016), regional SNP-based heritability approaches (Nagamine *et al.* 2012; Uemoto *et al.* 2013), and genomic prediction models using all available SNPs simultaneously (Meuwissen *et al.* 2001; Speed and Balding 2014). The main advantage of these methods is that they consider the contribution from SNPs whose effect sizes are too small to be classified as associated variants in a traditional GWAS.

One of the emerging themes obtained from GWAS is that top associated SNPs tend to cluster in biological pathways (Lango Allen *et al.* 2010; Lage *et al.* 2012; Maurano *et al.* 2012; O’Roak *et al.* 2012). This knowledge could be utilized more directly in the statistical models, and has the potential to increase the power to uncover the underlying biology of complex trait phenotypes. One approach is the statistical framework entitled genomic feature models, GFM (implemented as an R-package which is available at http://psoerensen.github.io/qgg/). We have successfully applied this modeling approach to cattle (Edwards *et al.* 2015; Fang *et al.* 2017a; Fang *et al.* 2017b), pigs (Sarup *et al.* 2016), mice (Ehsani *et al.* 2015), fruit flies (Edwards *et al.* 2016; Rohde *et al.* 2017; Sørensen *et al.* 2017) and humans (Rohde *et al.* 2016a), and have shown that these models can provide novel biological knowledge of complex traits. Some challenges with this approach still remain. First, when the genomic feature analysis is based on large gene sets, it may be useful to reduce, or restrict, the list of genes within the associated gene set, to those genes with the greatest contribution to the overall trait variability. Second, to date the results from GFM have been limited to discovery of putative causal variants, and true functional validation of the variants has been lacking.

We have previously described the SNP set test approach – the covariance association test (CVAT) – as a powerful method for associating a set of SNPs with human diseases and complex traits (Rohde *et al.* 2016a; Sørensen *et al.* 2017). Here, we propose that CVAT can be used to rank genes within a large gene set, which collectively display statistical association with the trait phenotype, according to their estimated effect sizes. In order to experimentally test this, we used *Drosophila melanogaster* as a model system. *D. melanogaster* has many advantages over other model systems, such as a short generation time, easy husbandry, limited ethical restrictions, and a vast diversity of readily available genetic tools (*e.g.*, functional mutants, temporal/spatial gene expression knockdown/in). A particularly useful resource is the *Drosophila* Genetic Reference Panel, DGRP (Mackay *et al.* 2012). The DGRP consists of 205 genome-wide homozygous lines derived by 20 generations of consecutive full-sib mating of wild-caught flies. Genome sequence data of the DGRP lines are publicly available (Mackay *et al.* 2012; Huang *et al.* 2014). The DGRP allows researchers to investigate the genetic basis for any quantitative trait phenotype. To date the DGRP has been used to study >45 quantitative traits (Anholt and Mackay 2017; Mackay and Huang 2017).

The aim of this study was twofold: (1) to investigate the applicability of CVAT to rank genes within a set of associated genes; and (2) to provide functional validation for the findings of the genomic feature models. First, we used the genomic feature prediction models to identify large sets of genes, here defined by gene ontology (GO) categories (The Gene Ontology Consortium 2000), that were predictive of the trait values. Next, we used CVAT to rank the genes, within the larger set of genes that when considered jointly increased the predictive performance, according to the genomic variance captured by the individual genes within the predictive GO term.

We applied these methods to the quantitative trait locomotor activity in *D. melanogaster*. Collecting data on a new phenotype instead of using published data has the advantage of allowing us to perform the functional validation in the same manner as we did in phenotyping the DGRP, as well as potentially providing new biological knowledge of a complex trait phenotype. Locomotion is an important fitness component that is central for an individual’s survival and reproduction because it allows animals to localize mates and energy resources, defend territories, and escape from predators and environmental stress elements. Locomotor activity is a complex trait, and the genetic component is governed by the joint segregation of multiple quantitative trait loci, and likely their interactions. As a measurable trait, locomotor activity encompasses a broad range of different types of activity measures, some of which are species specific. Despite species- and trait specific differences, quantitative genetic analyses have revealed abundant genetic variation for different measures of locomotor activity across species (Burnet *et al.* 1988; Swallow *et al.* 1998; Lightfoot *et al.* 2004, 2008, 2010; Turner *et al.* 2005; Jordan *et al.* 2006, 2007).

Many different aspects of *Drosophila* locomotion have been studied, including phototaxis, geotaxis (Carpenter 1905), circadian rhythms of locomotor activity (Konopka and Benzer 1971), and rover-sitter foraging behavior (Osborne 1997). *Drosophila* locomotor activity has been quantified in several different ways, such as reactivity methods (*i.e.*, quantifying the level of activity after physical disturbance) (Gargano *et al.* 2005; Jordan *et al.* 2006), infrared monitoring systems that quantify the number of times a fly passes a certain point (Rosato and Kyriacou 2006; Pfeiffenberger *et al.* 2010; Bahrndorff *et al.* 2016), and video tracking methods (Zimmerman *et al.* 2008; Colomb *et al.* 2012; Gilestro 2012; Garbe *et al.* 2015). Here, we quantified locomotor activity in the DGRP using a high-throughput video tracking method (Rohde *et al.* 2016b) to quantify the total distance covered during a five-minute trial.

## Methods

### Experimental design

**Figure 1:**
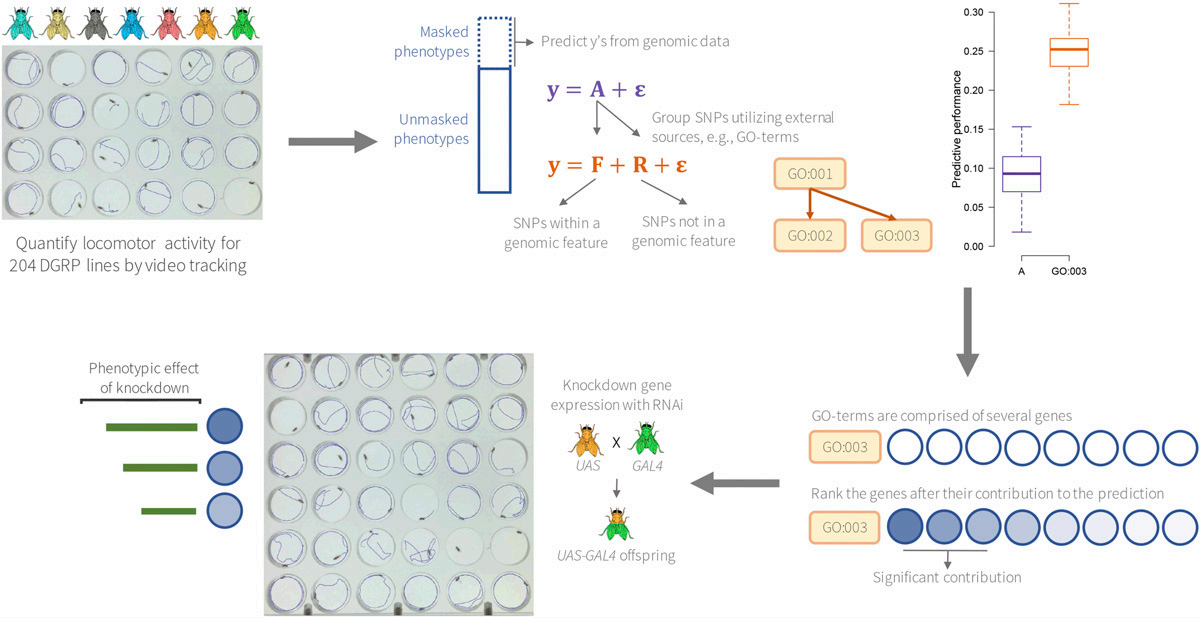
Outline of the study workflow starting with quantifying levels of activity for the 204 DGRP lines. A genomic feature prediction model was used to identify marker sets, defined by gene ontology (GO) terms that increased the predictive ability compared to a model where all markers were used simultaneously. The genes within a predictive GO term are likely to contribute unequally to the predictive performance, thus, the genes were ranked according to their contribution (quantified using CVAT). Top genes were functionally validated by suppressing gene expression and assessing the effect of such reduction on locomotor activity.

The workflow of this study is depicted in Figure 1. We quantified locomotor activity for 204 DGRP lines in a highly-replicated study design. Genomic feature sets were defined based on GO categories. Each feature set was used in a genomic prediction model, and the predictive performance was compared to a null model that weight all SNP markers equally. The genes within a particular GO category are likely to contribute unequally to the predictive performance, as well as to forming the trait phenotype. Therefore, we used CVAT to rank the genes within the predictive GO categories according to their contribution to the trait variation. The genes that contribute most within the predictive GO categories were selected, and used in a functional validation experiment, where expression of these genes was suppressed using the binary *UAS-GAL4* system, and the phenotypic consequence on locomotor activity was assessed.

### *Drosophila* stocks and husbandry

The DGRP lines (Mackay *et al.* 2012; Huang *et al.* 2014) were maintained in Prof. T. F. C. Mackay’s laboratory (North Carolina State University, Raleigh, North Carolina 27695) on cornmeal-molasses-agar medium, 25°, 70% humidity, and a 12-h light-dark cycle. *UAS*-RNAi lines for gene expression knockdown (*CG10920*^KK109327^, *CG14160*^KK104744^, *CG15553*^KK104514^, *CG1628*^KK109588^, *CG17930*^KK108912^, *CG32103*^KK108078^, *CG33233*^KK106897^, *Dic1*^KK103757^, *DPCoAC*^KK101378^, *Rim2*^KK100807^, *Shawn*^KK109948^) were obtained from the Vienna *Drosophila* Stock Center (http://stockcenter.vdrc.at) (Dietzl *et al.* 2007), and a *tubulin-GAL4* driver line (y^1^w*;P*{tubP-GAL4}*LL7*/TM3,Sb^1^*) was obtained from the Bloomington *Drosophila* Stock Center (http://flystocks.bio.indiana.edu). The *UAS*-RNAi and *GAL4* lines were maintained at the *Drosophila* laboratory at Department of Bioscience, Aarhus University (8000 Aarhus, Denmark) on oatmeal-yeast-sugar-agar medium, 25°, 70% humidity, and a 12-h light-dark cycle).

### Quantifying locomotor activity

We used an assay to quantify locomotor activity that relies on video tracking software (Rohde *et al.* 2016b). The DGRP lines were phenotyped at North Carolina State University (Raleigh, North Carolina 27695), and the *UAS-GAL4* RNAi knockdown lines were phenotyped at the Department of Bioscience, Aarhus University (8000 Aarhus, Denmark). Since phenotyping was performed in two different laboratories, there were small experimental differences between the two experimental designs (see details below).

#### Quantifying locomotor activity for the DGRP lines:

The behavioral arenas were constructed in transparent polycarbonate with 4 × 6 behavioral chambers (each of 16 mm in diameter and 6 mm in height) behavioral arena. The locomotor assays were performed in a behavioral room (25°, 70% relative humidity) between 8:00-11:00 am. The behavioral arenas were illuminated with exogenous light sources. An iPad Mini (Apple, Cupertino, California) was mounted above the behavioral arena to obtain five minutes of video recordings from each of the 24 behavioral chambers. Locomotor activity was then quantified as the total distance traveled for individual flies, extracted from the video files using the tracking software EthoVision XT (v. 10.0) (Noldus, Wageningen, The Netherlands).

We obtained locomotor activity measurements for approximately 24 individual males for each of 204 DGRP lines. For logistical reasons the DGRP lines were divided into eight blocks, such that each block contained approximately 28 DGRP lines, and every block was assayed over six consecutive days. Two to five day old flies were anesthetized with CO_2_ and transferred to the behavioral arenas 16-18 hr prior to the assay. During this time, the flies had access to food, which was removed at the start of the assay.

#### Quantifying locomotor activity for the gene expression knockdown lines:

The behavioral arenas used for the gene expression knockdown experiments were likewise constructed in transparent polycarbonate, but contained 6 × 6 behavioral chambers (each of 16 mm in diameter and 6 mm in height) per behavioral arena. The behavioral arenas were illuminated from below by a light box (LP400, Dörr, Chesterfield, UK) to ensure high contrast between the flies and the background. An iPad Air (Apple, Cupertino, California) mounted above the behavioral arena was used to obtain ten minutes of video recordings. All behavioral tests were performed in a behavioral room (25°, 70% relative humidity) between 8:00-11:00 am.

For each *UAS*-RNAi line, and the corresponding control line, approximately 20 virgin females were crossed to five *tubulin-GAL4* males. Approximately 30 F1 male offspring containing the *UAS-GAL4* construct were assayed. The flies being tested were gently moved to the individual behavioral chambers (without anesthetization using an aspirator), and the video recordings were obtained immediately after loading the flies to the behavioral arenas. The observed phenotype of the *UAS-GAL4* offspring was compared to the offspring of the control line crossed to the *GAL4* line using a standard linear model accounting for experimental effects (date and behavioral plates).

### Quantitative genetic analysis

To estimate the broad sense heritability ($H^{2}$) of locomotor activity in the DGRP, we fitted the mixed model Y=μ+d+b+p+L+E, where Yo is the phenotype, μ is the overall mean, d is the fixed effect to account for different measurement days, b is a fixed block effect, p is a fixed plate effect, L is the random line effect, and E is the residual. The broad sense heritability was estimated as ${\hat{H}}^{2}=\frac{{\hat{\sigma }}_{L}^{2}}{{\hat{\sigma }}_{L}^{2}+{\hat{\sigma }}_{E}^{2}}$, where ${\hat{\sigma }}_{L}^{2}$ and ${\hat{\sigma }}_{E}^{2}$ are the variance components for the line and residual terms. Variance components were estimated using the *lme4* package for R (Bates *et al.* 2015; R Core Team 2017).

### Genomic feature models

The following section describes the workflow for genomic prediction utilizing prior biological knowledge. The first step is to link SNPs to the genomic feature classes. The second steps involve computing genomic relationship matrices and performing the prediction. Finally, the genomic variance within predictive feature sets was partitioned to minor units, such as genes. Functions and example scripts are publicly available at http://psoerensen.github.io/qgg/.

#### Genomic data and feature sets:

The DGRP genotypes were obtained from http://dgrp2.gnets.ncsu.edu/. All genomic analyses were based on segregating biallelic SNPs obtained using the standard filtering process (Mackay *et al.* 2012; Huang *et al.* 2014): SNPs were included if the minor allele frequency ≥0.05, if the Phred quality score (the sequencing quality of a given SNP) >500, and if the genotype call rate ≥0.8. This resulted in 1,725,755 SNPs distributed across the six chromosome arms (*2R*, *2L*, *3R*, *3L*, *4* and *X*).

The feature sets considered were genes and gene ontology (GO) categories (The Gene Ontology Consortium 2000). First, SNPs were mapped to genes (all SNPs within the open reading frame) using FlyBase v5.49 annotations of the *D. melanogaster* reference genome (Tweedie *et al.* 2009). Second, genes were aggregated based on gene ontology categories using the BioConductor package *org.Dm.eg.db* (Carlson 2015). A total of 963,235 SNPs was mapped to 10,517 known genes and 1,134 GO terms. The number of SNPs within a single GO term varied from 23 to 163,938 SNPs.

#### Additive genomic relationship matrices:

A central component for predicting trait values using genomic best linear unbiased prediction (GBLUP) is a matrix that captures the genetic similarity between all pairs of individuals. The additive genomic relationship matrix can be computed as $G=WW^{'}/m$ (VanRaden 2008), where m is the number of SNPs on which the relationship matrix is computed, and W is a centered and scaled genotype matrix. Each column vector of W is computed as $w_{i}=\frac{a_{i}-2p_{i}}{\sqrt{2p_{i}(1-p_{i})}}$, where $p_{i}$ is the minor allele frequency of the *i*-th SNP, and $a_{i}$ is the i-th column vector of the allele count matrix, A, that contains the genotypes coded as 0 and 2 (counting the number of minor alleles).

The common use of GBLUP models is to model a single random genomic effect, thus, assuming that all SNP effect sizes are drawn from a common Gaussian distribution. If including multiple random genomic effects, this assumption can be relaxed by allowing SNP effect sizes to have different distributions. Incorporating multiple random effects requires the computation of additional genomic relationship matrices based on a subset of SNPs, for example, those within a genomic feature (f) and the remaining SNPs not within the feature set (r); $G_{f}=W_{f}W_{f}^{’}/m_{f}$ and $G_{r}=W_{r}W_{r}^{’}/m_{r}$.

#### Genomic prediction:

In the general case, the GBLUP (Meuwissen *et al.* 2001) model is written as

y=Xb+Zg+e. (Model 1)

where y is a vector of phenotypic observations, X and Z are design matrices linking fixed (b) and random genomic effects (g) to the observations, and the residual effects (e). Under this model, it is assumed that the observed phenotype is y∼N(Xb, V) where $V=ZGZ’\sigma_{g}^{2}+I\sigma_{e}^{2}$. A commonly used method to assess the predictive performance is to apply a cross-validation scheme, where a subset of the data are masked. To avoid undesirable data structure in the resampling, it may be helpful to adjust the phenotypes for fixed effects. Model 1 is an animal model with repeated measurements per DGRP line, thus, to retain the replicate data structure the adjusted phenotypic values for the i-th DGRP line was computed as ${\tilde{y}}_{i}={\hat{g}}_{i}+{\hat{e}}_{i}$ (one $\hat{g}$ per DGRP line, and one $\hat{e}$ per DGRP line per replicate). Thus, the GBLUP model reduces to

$$\tilde{y}=Zg+e.\text{ (Model 2)}$$

When Model 2 has been fitted on the training data (t), the genomic effects in the validation set (v) can be computed using Equation 1,

$${\hat{g}}_{v}=\left(G_{v,t}{\hat{\sigma }}_{g}^{2}\right){\left[G_{t,t}{\hat{\sigma }}_{g}^{2}+I_{t,t}{\hat{\sigma }}_{e}^{2}\right]}^{-1}\left({\tilde{y}}_{t}-{\hat{\mu }}_{t}\right)\text{ (Equation 1)}$$

Model 2 can be extended to a genomic feature model (GFBLUP, Model 3) by dividing the total genomic effects captured by all SNPs, by the genomic effects captured by SNPs within the feature set (f), and the genomic effects captured by the SNPs not included in the feature set (r) (Edwards *et al.* 2015, 2016; Rohde *et al.* 2017; Sørensen *et al.* 2017),

$$\tilde{y}=Zf+Zr+e.\text{ (Model 3)}$$

The total genomic effects in the validation set can then be computed using Equation 2,

$${\hat{g}}_{v}=\left(G_{f_{v,t}}{\hat{\sigma }}_{f}^{2}+G_{r_{v,t}}{\hat{\sigma }}_{r}^{2}\right){\left[G_{f_{t,t}}{\hat{\sigma }}_{f}^{2}+G_{r_{t,t}}{\hat{\sigma }}_{r}^{2}+I_{t,t}{\hat{\sigma }}_{e}^{2}\right]}^{-1}\times \left({\tilde{y}}_{t}-{\hat{\mu }}_{t}\right).\text{ (Equation 2)}$$

The predictive performance (PA) of the GBLUP and GFBLUP models was quantified as Pearson’s correlation between the observed and predicted genomic values. The models (Model 2 or Model 3) were fitted using 90% of the data, and the estimated genomic parameters were used to predict the genomic values in the remaining 10% of the data. This procedure was repeated 50 times on random subdivisions of the entire data set. This prediction design was chosen as similar prediction studies using the DGRP system has been found usable (Rohde *et al.* 2017; Sørensen *et al.* 2017). A genomic feature model (Model 3) was fitted for each of the genomic feature categories, and the predictive performance of each genomic feature model was compared to the null model (Model 2) by assessing if the predictive ability of the genomic feature model was increased compared to the GBLUP model using Welch’s *t*-test of unequal variance (Welch 1947). Subsequently, all *p*-values were adjusted for multiple testing using the false discovery rate, and significance level was set to 0.05.

In addition to evaluating the models on predictive performance, the GBLUP and GFBLUP models were also assessed based on estimated genomic parameters. Inferences on the genomic heritability of the models were based on ${\hat{h}}_{\text{GBLUP}}^{2}={\hat{\sigma }}_{g}^{2}/\left({\hat{\sigma }}_{g}^{2}+{\hat{\sigma }}_{e}^{2}\right)$, and ${\hat{h}}_{\text{GFBLUP}}^{2}=\left({\hat{\sigma }}_{f}^{2}+{\hat{\sigma }}_{r}^{2}\right)/\left({\hat{\sigma }}_{f}^{2}+{\hat{\sigma }}_{r}^{2}+{\hat{\sigma }}_{e}^{2}\right)$, as well as by partitioning the genomic variance of the GFBLUP model ${\hat{h}}_{f}^{2}=\left({\hat{\sigma }}_{f}^{2}\right)/\left({\hat{\sigma }}_{f}^{2}+{\hat{\sigma }}_{r}^{2}\right)$ and ${\hat{h}}_{r}^{2}=\left({\hat{\sigma }}_{r}^{2}\right)/\left({\hat{\sigma }}_{f}^{2}+{\hat{\sigma }}_{r}^{2}\right)$. These ratios quantify the proportion of total genomic variance captured by (${\hat{h}}_{f}^{2}$), and not captured by (${\hat{h}}_{r}^{2}$), the SNPs in the feature set.

Estimating the variance components in Model 2 and Model 3 was performed using the average information restricted maximum likelihood (AI-REML) procedure (Madsen *et al.* 1994; Johnson and Thompson 1995) as implemented in DMU software. We have developed an R interface that enables users to perform analysis within R that otherwise rely on DMU (DMU can be downloaded from http://dmu.agrsci.dk/DMU/). Our R package qgg is accessible at http://psoerensen.github.io/*qgg*/, including examples on how to perform the genomic feature analyses.

#### Partitioning of genomic variance to gene level:

To partition the genomic variance of a predictive GO category to genomic variance at the gene level we adapted the covariance association test (CVAT) (Rohde *et al.* 2016a; Sørensen *et al.* 2017).

The CVAT method was originally developed as a set test approach that captures the covariance between the total genomic effects from all markers and the genomic effects from the markers within the feature set (Rohde *et al.* 2016a). Here, we instead considered the covariance between the genomic effects of a GO term (${\hat{g}}_{\text{GO}}$) and the genomic effects at gene level within that particular GO term (${\hat{g}}_{\text{gene}}$),

$$T_{CVAT}={\hat{g}}_{\text{GO}}{\hat{g}}_{\text{gene}},\text{ (Equation 3)}$$

where ${\hat{g}}_{\text{GO}}$ are the genomic feature effects estimated from Model 3, and ${\hat{g}}_{\text{gene}}=\overset{m_{\text{gene}}}{\underset{i=1}{\sum }}w_{i}{\hat{s}}_{{\text{GO}}_{i}}$. The vector of SNP effects, ${\hat{s}}_{{\text{GO}}_{i}}=W_{\text{GO}}^{’}{\left(W_{\text{GO}}W_{\text{GO}}^{’}\right)}^{-1}\;{\hat{g}}_{\text{GO}}$, where $W_{\text{GO}}$ corresponds to the centered and scaled genotype matrix of the SNPs within one particular GO term. To determine the degree significance an empirical distribution of $T_{CVAT}$ was obtained based on a circular permutation approach (Cabrera *et al.* 2012), where the genome was considered circular in order to retain the same order of SNPs but receive new SNP effects in each permutation. This decouples the association between the SNP and the genomic feature, but retains the correlation structure among the SNP effects. In each iteration of the permutation approach a new $T_{CVAT}$ statistic was computed (repeated 10,000 times), and the *p*-value was computed as a one-tailed test of the proportion of the randomly sampled summary statistics being larger than the observed summary statistic (see Rohde *et al.* (2016a) and Sørensen *et al.* (2017) for additional details).

### Marginal SNP analysis

The CVAT results were compared to a standard marginal SNP analysis. Single marker associations evaluate the association between each segregating SNP and the trait variation. In order to account for the experimental fixed effects and the genetic similarity among DGRP lines the estimated genomic effects ($\hat{g}$, from Model 2) was used as response variable. The marginal SNP analysis was a *t*-test on the regression coefficient from the regression of $\hat{g}$ on each segregating SNP in the DGRP, *i.e.*, a total of 1,725,755 regression analyses.

### Data availability

The DGRP genotypes can be accessed via the DGRP2 website http://dgrp2.gnets.ncsu.edu/, and the phenotypic data are given in Table S1. Supplemental material available at Figshare: https://doi.org/10.25387/g3.5951581.

## Results and Discussion

**Figure 2:**
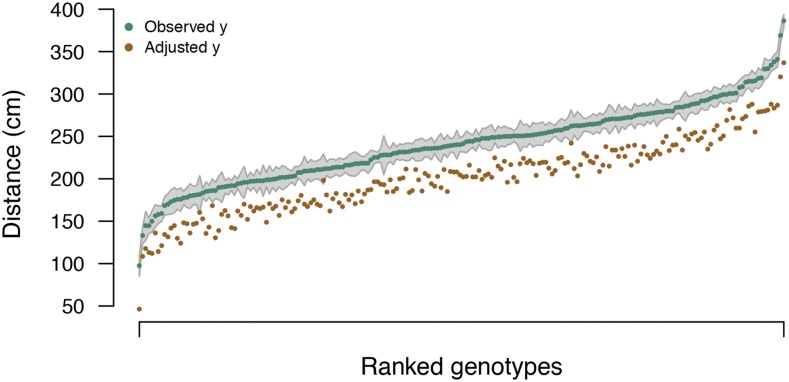
Rank-ordered mean for locomotor activity (green) and the corresponding mean values adjusted for experimental fixed effects (brown). Shaded area depicts the standard error of the mean.

We quantified male locomotor activity in 204 DGRP lines using video tracking to measure the total distance traveled per individual in the course of five minutes. We found substantial genetic variation (Table S2) in locomotor activity, with an approximate fourfold difference between the least active and the most active DGRP lines (Figure 2, Table S1). The broad sense heritability for male locomotor activity was ${\hat{H}}^{2}=0.40$ (SE = 0.03). We estimated the proportion of total phenotypic variation explained by common variants (MAF>0.05) using the additive genomic relationship matrix as ${\hat{h}}^{2}=0.26$ (SE = 0.02); thus, 65% (0.26 / 0.40) of the total genetic variation was captured by common, additive variants. The estimated broad sense heritability is in the range of other estimates of *D. melanogaster* locomotor activity (Jordan *et al.* 2006, 2007).

We used the GBLUP model (Model 2) to predict the genomic values for locomotor activity by estimating genomic parameters on 90% of the data, and using those parameters to predict the genomic values in the remaining 10% of the data (Equation 1). The validation sets were chosen randomly and this procedure was repeated a total of 50 times on different training and validation sets. The GBLUP model uses all available SNPs assuming the effects are drawn from a common Gaussian distribution. The performance of the model was quantified as the correlation (r) between the predicted and observed phenotypic values in the validation set. We found low mean predictive ability (PA ± SEM) for the GBLUP model (PA = 0.12 ± 0.033). The maximum predictive ability of line means is $H^{2}=r^{2}={\text{PA}}^{2}$ (Mrode 2005; Goddard 2009). The heritability based on line means can be approximated as ${\hat{H}}^{2}={\hat{\sigma }}_{g}^{2}/\left({\hat{\sigma }}_{g}^{2}+\frac{{\hat{\sigma }}_{e}^{2}}{\bar{n}}\right)$ (Mackay and Huang 2017), where ${\hat{\sigma }}_{g}^{2}$ and ${\hat{\sigma }}_{e}^{2}$, respectively, are the among-line and within-line variance of the individual data, and $\bar{n}$ is the average number of flies scored per DGRP line (here $\bar{n}=23$). The broad sense heritability of line means was ${\hat{H}}^{2}=0.94$, thus, the GBLUP model only accounts for 0.014/0.94 = 1.5% of the observed heritability of line means. Thus, assuming all SNP effects to be from a common Gaussian distribution resulted in a low proportion of the heritability explained, in agreement with a similar study on *D. melanogaster* aggressive behavior (Rohde *et al.* 2017).

The one component GBLUP model assumes all SNP effects are from a common Gaussian distribution; however, this assumption is not likely to be true (Speed and Balding 2014). A relaxation of the Gaussian assumption can be obtained by fitting multiple random components, such as the GFBLUP models (Model 3), by allowing the SNP effects within those components to have different effect sizes (small-moderate-large). An example of this was shown by Rohde *et al.* (2016a), where the SNPs were partitioned according to minor allele frequencies to obtain different distributions of SNP effects. Here, we build genomic relationship matrices based on SNPs within GO categories. For each GO term the GFBLUP model was fitted, and the predictive abilities were compared to the predictive performance of the GBLUP model. The five GO terms with the highest predictive abilities are shown in Table 1 (the full list is given in Table S3).

**Table 1:** The top five GO terms with highest predictive ability (PA). For each GO term the following information is listed: Number of genes (No. genes) and SNPs (No. SNPs) within the GO term, the mean PA with standard errors (SE), the raw (*p*) and adjusted *p*-values (by false discovery rate (FDR)) for increased predictive performance compared to the GBLUP model, and the proportion of genomic variance explained by the GO term ($h_{f}^{2}$)

**GO term**	**No. genes**	**No. SNPs**	**PA ± SE**	***p*-value**	**FDR *p*-value**	***h*_*f*_^2^**
1. GO:0022857	59	2563	0.35 ± 0.026	$6.8\times 10^{-6}$	$7.7\times 10^{-5}$	0.53
2. GO:0006730	17	749	0.27 ± 0.029	$2.1\times 10^{-4}$	$1.2\times 10^{-1}$	0.28
3. GO:0006810	80	6893	0.25 ± 0.028	$9.5\times 10^{-4}$	$3.6\times 10^{-1}$	0.44
4. GO:0055114	368	22029	0.25 ± 0.029	$1.7\times 10^{-3}$	$3.9\times 10^{-1}$	1.00
5. GO:0030866	21	2161	0.25 ± 0.027	$1.3\times 10^{-3}$	$3.7\times 10^{-1}$	0.30

1: transmembrane transporter activity; 2: one-carbon metabolic process; 3: transport; 4: oxidation-reduction process; 5: cortical actin cytoskeleton organization.

**Figure 3:**
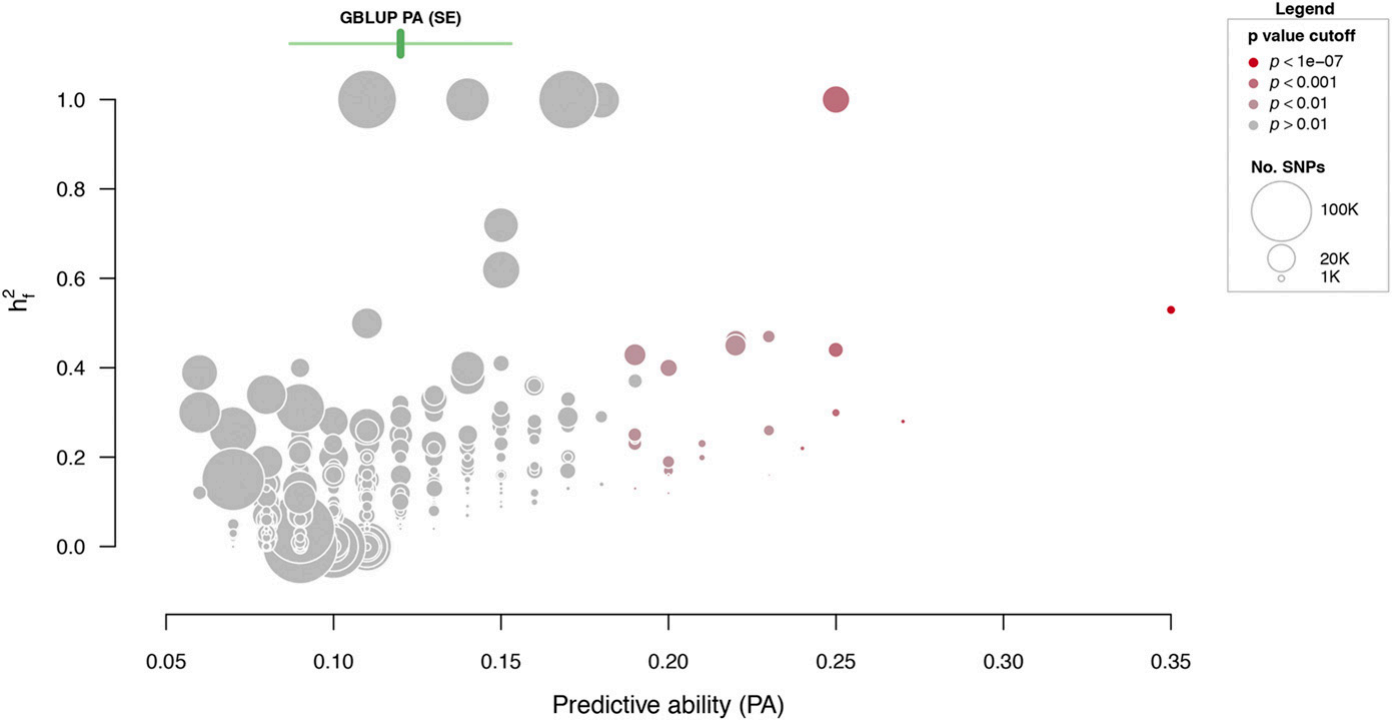
Results from the GFBLUP models. Each point corresponds to one GO term that is plotted within the space of genomic variance explained ($h_{f}^{2}$) and predictive ability (PA). The size of each point relates to the number of SNPs within the GO term, and the color indicates the *p*-value of increased predictive ability compared to the GBLUP model. The mean predictive ability ± standard error (SE) of the GBLUP model is indicated with green vertical and horizontal lines, respectively.

When we jointly examined several model parameters, interesting patterns emerged (Figure 3). GO terms with high PA also tended to explain a larger fraction of the genomic variance ($h_{f}^{2}$). GO terms with many SNPs do not have higher predictive abilities, or capture more of the genomic variance. Instead, large GO categories, *i.e.*, those that contain many SNPs, tend to explain the least genomic variance (Figure 3). This is probably a consequence of too many non-causal SNPs in the feature set, which adds noise to the model. Four non-significant and one marginally significant GO term explain 100% of the genomic variance (Figure 3). Explaining 100% of the variance when the analysis is based on a small proportion of all genomic markers is naturally an overestimation. This can arise if two genomic relationship matrices are very similar, because then it is likely that parts of the genomic variance will be captured by only one of the components, thereby leading to overestimation.

Next, we considered the GO term that increased the predictive ability significantly compared to the GBLUP model in more detail (mean PA = 0.35 ± 0.026, Table 1). The predictive GO term GO:0022857 contains genes involved in transmembrane transport. In the DGRP genotype data the GO term GO:0022857 contained 59 genes and 2,563 biallelic SNPs (at MAF > 0.05). Partitioning the genomic variance between the SNPs within GO:0022857 and SNPs not located within genes related to GO:0022857, the GFBLUP model accounts for 13% of the heritability compared to the standard GBLUP model that only accounted for 1.5%. Thus, allowing for differential weight on the SNP effects increased the predictive performance, and therefore increased how much of the heritability the GFBLUP model accounted for. This pattern is similar to the observation in Rohde *et al.* (2017) for aggressive behavior in the DGRP. We then partitioned the genomic variance within that GO term among the 59 genes using CVAT. This method considers the covariance between the total genomic effects of the GO term and the genomic effects of the genes within the GO term (Equation 3). The resulting statistic is a *p*-value indicating if the proportion of genomic variance explained by the gene is larger than a randomly sampled set of SNPs containing the same number of SNPs as the gene being considered. A total of 15 genes had a *p*-value < 0.05, indicating that these genes capture a larger proportion of the total genomic variance within the predictive GO term than a random set of SNPs within that GO term (Table S4). We compared this result with the results from the marginal SNP analysis where no SNPs passed the genome-wide significance threshold (Figure S1). The SNP *p*-values of the genetic markers located within the 15 CVAT associated genes ranged from $1.45\times 10^{-2}$ to $4.35\times 10^{-6}$. The majority of the CVAT associated genes had SNP *p*-values around $1\times 10^{-3}$ (Figure S1); thus, these genes would not have been identified by the marginal SNP analysis. This discrepancy in results was expected because the marginal SNP analysis picks up individual SNPs with the largest effects, whereas CVAT evaluates the joint effect of multiple genomic markers and can therefore detect SNPs that individually have small effects.

**Figure 4:**
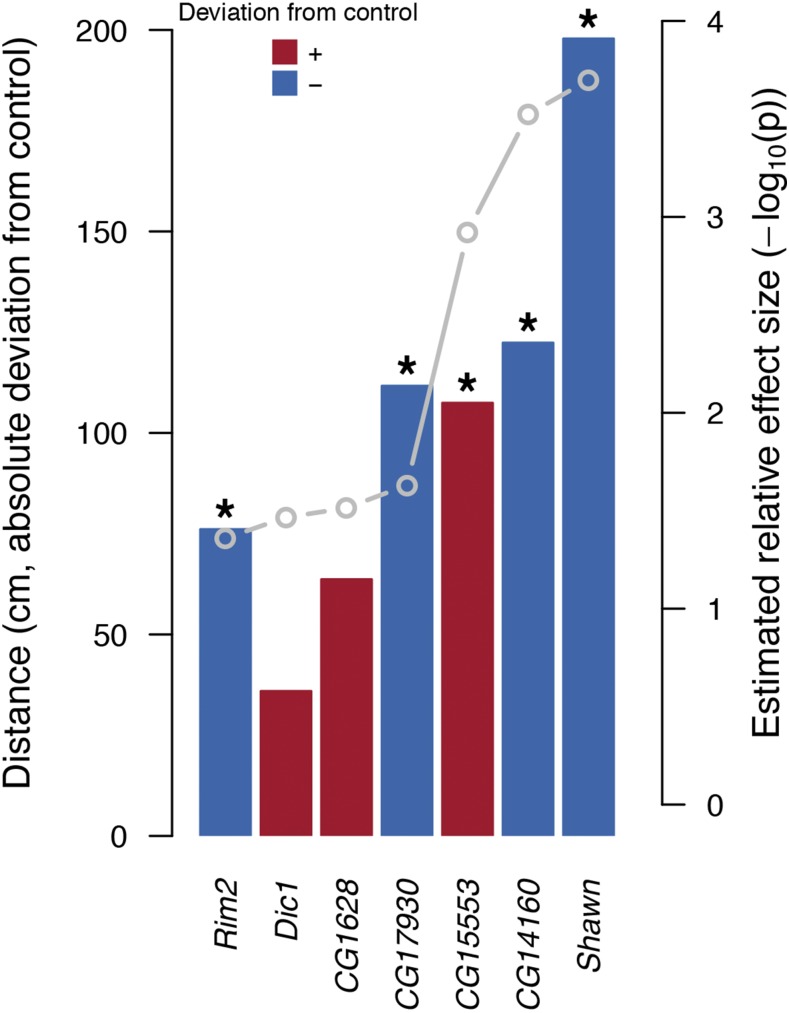
Effects on distance moved by gene expression knockdown expressed as absolute deviations from control line (red: increased activity; blue: decreased activity) ranked according to estimated effect size. Asterisks (∗) indicate statistical significance (*i.e.*, *p* < 0.05) between knockdown strain and control strain. Gray points (belongs to the right y-axis) show the estimated relative effect size within the predictive GO term, here illustrated as −log10(*p*) from CVAT.

Given our list of 15 candidate genes potentially affecting locomotor activity, we set out to functionally validate these genes by investigating the phenotypic consequence of gene expression knockdown in adult flies using the bipartite *UAS*-GAL4 system. Only 11/15 genes were available with the desired genetic background, of which seven lines produced viable offspring after crossing to the ubiquitous *GAL4*-driver. Thus, a total of seven genes were assessed for their effect of gene expression knockdown on locomotor activity; *CG1628*, *CG14160*, *CG15553*, *CG17930*, *Dic1*, *Rim2* and *Shawn*. Five of the seven tested knockdown lines resulted in significant locomotor deviations from the control line with the same genetic background (Figure 4). The gene expression knockdown resulted in offspring becoming both more (*CG15553*) and less active (*Rim2*, *CG17930*, *CG14160*, *Shawn*, Figure 4) than the respective control line, indicating that the knockdown lines do not in general suffer strongly from the gene expression knockdown. Importantly, the correlation between the absolute effect size of gene expression knockdown and degree of genomic variance explained was very high, ρ=0.91 (*p*-value = 0.005, Figure 4). Thus, we not only validated the functional effects of the candidate genes for locomotor activity, but also provided functional evidence supporting the success of our method for identifying a restricted set of important genes ranked by their effect sizes from a larger set of potential candidate genes.

The genes *CG14160*, *CG15553*, and *CG17930* have not previously been phenotypically annotated in *D. melanogaster*, thus, here we provide first evidence that the genes are involved in explaining variation in a behavioral phenotype. The gene *Shawn* is a mitochondrial carrier in the *Drosophila* nervous system (Slabbaert *et al.* 2016), and *Rim2* encodes a deoxynucleotide transporter located within the mitochondria. Both *Rim2* and *Shawn* have conserved human homologous gene sequences, *SLC25A36* and *SLC25A40*, respectively, which have been found to contain susceptibility loci for bipolar disorder (Winham *et al.* 2014) and epilepsy (Sirén *et al.* 2010).

Fruit flies and mammals have a common evolutionary origin of basic biological processes, including development of the nervous system (Adams *et al.* 2000), and approximately 75% of human disease genes have at least one homologous gene in *D*. *melanogaster* (Reiter *et al.* 2001). Human neurological diseases, *e.g.*, Parkinson’s and Huntington’s diseases, are associated with locomotor deficits, whereas some neuropsychiatric disorders, *e.g.*, attention-deficit/hyperactivity disorder and depression, are associated with changes in activity levels (American Psychiatric Association 2013). Therefore, understanding the genetic architecture of locomotor activity in model organisms might also provide an important link to human health.

For example, Parkinson’s disease has been shown to be linked to degeneration of certain dopaminergic neurons (Olanow and Tatton 1999), and dopamine has been shown to affect locomotion in fruit flies (Connolly *et al.* 1971; Jordan *et al.* 2006; Riemensperger *et al.* 2011; van der Voet *et al.* 2015), and mice (Garland *et al.* 2011). The fact that both *Rim2* and *Shawn* have conserved human homologous gene sequences, *SLC25A36* and *SLC25A40*, respectively, which have been found to contain susceptibility loci for bipolar disorder (Winham *et al.* 2014) and epilepsy (Sirén *et al.* 2010) illustrate the potential of using *D. melanogaster* as a model organism to study complex human psychological and behavioral disorders.

In conclusion, we provide functional support both for the candidate genes detected by CVAT, and for the ranking of effect sizes suggested by CVAT. These results are important because they provide evidence for the two challenges relating to GFM analyses; namely the need to have an efficient method to rank genes within a larger set of associated genes, and to perform biological validation of the genomic findings from GFM. Thus, these results demonstrate that the findings from the GFM analyses not are statistical artifacts, but indeed have biological relevance.
